# Synthesis and Behavior of Cetyltrimethyl Ammonium Bromide Stabilized Zn_1+x_SnO_3+x_ (0 ≤ x ≤1) Nano-Crystallites

**DOI:** 10.1371/journal.pone.0156246

**Published:** 2016-05-26

**Authors:** Astrid Placke, Ashok Kumar, Shashank Priya

**Affiliations:** 1 Department of Physics, National Institute of Technology Kurukshetra, Haryana, 136119, India; 2 Department of Physical Technologies, University of Applied Sciences and Art, Göttingen, 37083, Germany; 3 Center for Energy Harvesting Materials and Systems (CEHMS), Virginia Tech, Blacksburg, Virginia, 24061, United States of America; Institute for Materials Science, GERMANY

## Abstract

We report synthesis of cetyltrimethyl ammonium bromide (CTAB) stabilized Zn_1+x_SnO_3+x_ (0 ≤ x ≤1) nano-crystallites by facile cost-effective wet chemistry route. The X-ray diffraction patterns of as-synthesized powders at the Zn/Sn ratio of 1 exhibited formation of ZnSn(OH)_6_. Increasing the Zn/Sn ratio further resulted in the precipitation of an additional phase corresponding to Zn(OH)_2_. The decomposition of these powders at 650°C for 3h led to the formation of the orthorhombic phase of ZnSnO_3_ and tetragonal SnO_2_-type phase of Zn_2_SnO_4_ at the Zn/Sn ratio of 1 and 2, respectively, with the formation of their mixed phases at intermediate compositions, i.e., at Zn/Sn ratio of 1.25, 1.50 and 1.75, respectively. The lattice parameters of orthorhombic and tetragonal phases were a ~ 3.6203 Å, b ~ 4.2646 Å and c ~ 12.8291Å (for ZnSnO_3_) and a = b ~ 5.0136 Å and c ~ 3.3055Å (for Zn_2_SnO_4_). The transmission electron micrographs revealed the formation of nano-crystallites with aspect ratio ~ 2; the length and thickness being 24, 13 nm (for ZnSnO_3_) and 47, 22 nm (for Zn_2_SnO_4_), respectively. The estimated direct bandgap values for the ZnSnO_3_ and Zn_2_SnO_4_ were found to be 4.21 eV and 4.12 eV, respectively. The ac conductivity values at room temperature (at 10 kHz) for the ZnSnO_3_ and Zn_2_SnO_4_ samples were 8.02 × 10^−8^ Ω^-1^ cm^-1^ and 6.77 × 10^−8^ Ω^-1^ cm^-1^, respectively. The relative permittivity was found to increase with increase in temperature, the room temperature values being 14.24 and 25.22 for the samples ZnSnO_3_ and Zn_2_SnO_4_, respectively. Both the samples, i.e., ZnSnO_3_ and Zn_2_SnO_4_, exhibited low values of loss tangent up to 300 K, the room temperature values being 0.89 and 0.72, respectively. A dye-sensitized solar cell has been fabricated using the optimized sample of zinc stannate photo-anode, i.e., Zn_2_SnO_4_. The cyclic voltammetry revealed oxidation and reduction around 0.40 V (current density ~ 11.1 mA/cm^2^) and 0.57 V (current density– 11.7 mA/cm^2^) for Zn_2_SnO_4_ photo-anode in presence of light.

## Introduction

The synthesis of various single cation oxides, e.g., ZnO, TiO_2_, SnO_2_, MgO, NiO, Fe_2_O_3_, Nb_2_O_3_ has been reported by numerous techniques such as sol-gel, hydrothermal, solvo-thermal, solid state reaction, thermal evaporation, co-precipitation, etc. These materials have been used in several applications viz., Li-ion batteries, dye-sensitized solar cells (DSSCs), gas sensors, and photocatalysts for water splitting and organic pollutant degradation [1–11]. The multi-cation oxides have emerged as a potential alternative, and yet rarely been explored for optical devices such as DSSC [4, 12–13]. The perovskite family, i.e., ABO_3_ and A_2_BO_4_ being of special interest because of being chemically, thermally and mechanically stable wide bandgap semiconductor, which can provide high optical transmittance, reduced photo-bleaching, reduced electron-triiodide back recombination rate, and high open circuit voltage [1, 14–16]. Particularly, interest in the zinc stannate (ZnSnO_3_ and Zn_2_SnO_4_) nano-crystallites has been surged recently due to their important optical, electrochemical and photoelectrochemical properties and various technological applications in several devices such as light emitting diodes, solar cells, and biosensors [1, 17–20].

Multi-cation material provides flexibility to engineer its physical and/ or chemical behavior by varying the composition [21]. The n-type bi-cation transparent conducting oxide such as ZnO-In_2_O_3_ has revealed change in its work function, bandgap energy, resistivity and acid etching rate as the function of Zn/In content [22]. The abundance and tunable behavior make these multi-cation compounds interesting for continued research. The ZnSnO_3_, Zn_2_SnO_4_ are wide bandgap n-type ternary semiconductor oxides with better corrosion resistance, faster charge injection and faster electron diffusion efficiency than anatase- TiO_2_ used in conventional dye-sensitized solar cell. The ZnSnO_3_, Zn_2_SnO_4_ and/or intermediate mixed nano-crystalline phases are formed depending upon the Zn/Sn molar ratio of precursor compounds [1]. In general ZnSnO_3_ precipitates in orthorhombic phase, while Zn_2_SnO_4_ precipitates in cubic- spinel –type phase [14, 23]. In ZnSnO_3_, Zn^2+^ and Sn^4+^ cations are distributed at tetrahedral and octahedral sites, respectively, while in Zn_2_SnO_4_, Zn^2+^ are distributed equally at tetrahedral and octahedral sites, respectively, while Sn^4+^ cations occupy the octahedral sites.

Limited number of studies are available in literature on utilizing Al_2_O_3_, NiO, ZnO and graphene/ TiO_2_ for sensitization of perovskite solar cell [24]. The ternary oxides such as BaSnO_3_, ZnSnO_3_ and Zn_2_Sn_2_O_4_ have rarely been investigated [4, 24]. In this paper, we report the synthesis of cetyltrimethyl ammonium bromide (CTAB) stabilized ZnSnO_3_, Zn_2_SnO_4_ nano-crystallites and their intermediate compositions by facile cost-effective wet chemistry route. These nano-crystallites have been investigated for their structural, optical, dielectric and ac conductivity behavior. Here, an attempt has been made to synthesize tetragonal SnO_2_-type phase of Zn_2_SnO_4_, which has not normally been reported [25]. Many reports have revealed synthesis of ZnSnO_3_, Zn_2_SnO_4_ nanostructures of various shapes, e.g., sphere, cube, anisotropic rods, etc. by the use of mineralizers and additives [16]. In present investigation, the particle size and nano-crystallite- type morphology has been realized using surfactant CTAB, which is vital for high absorption of dye and faster electron transport. The facile wet chemistry route utilized for the synthesis has advantage of precisely controlling thermodynamics and kinetic of nucleation and growth of nano-crystallites, which is otherwise difficult to control with other synthesis routes [16, 26].

## Materials and Methods

**Fig 1:**
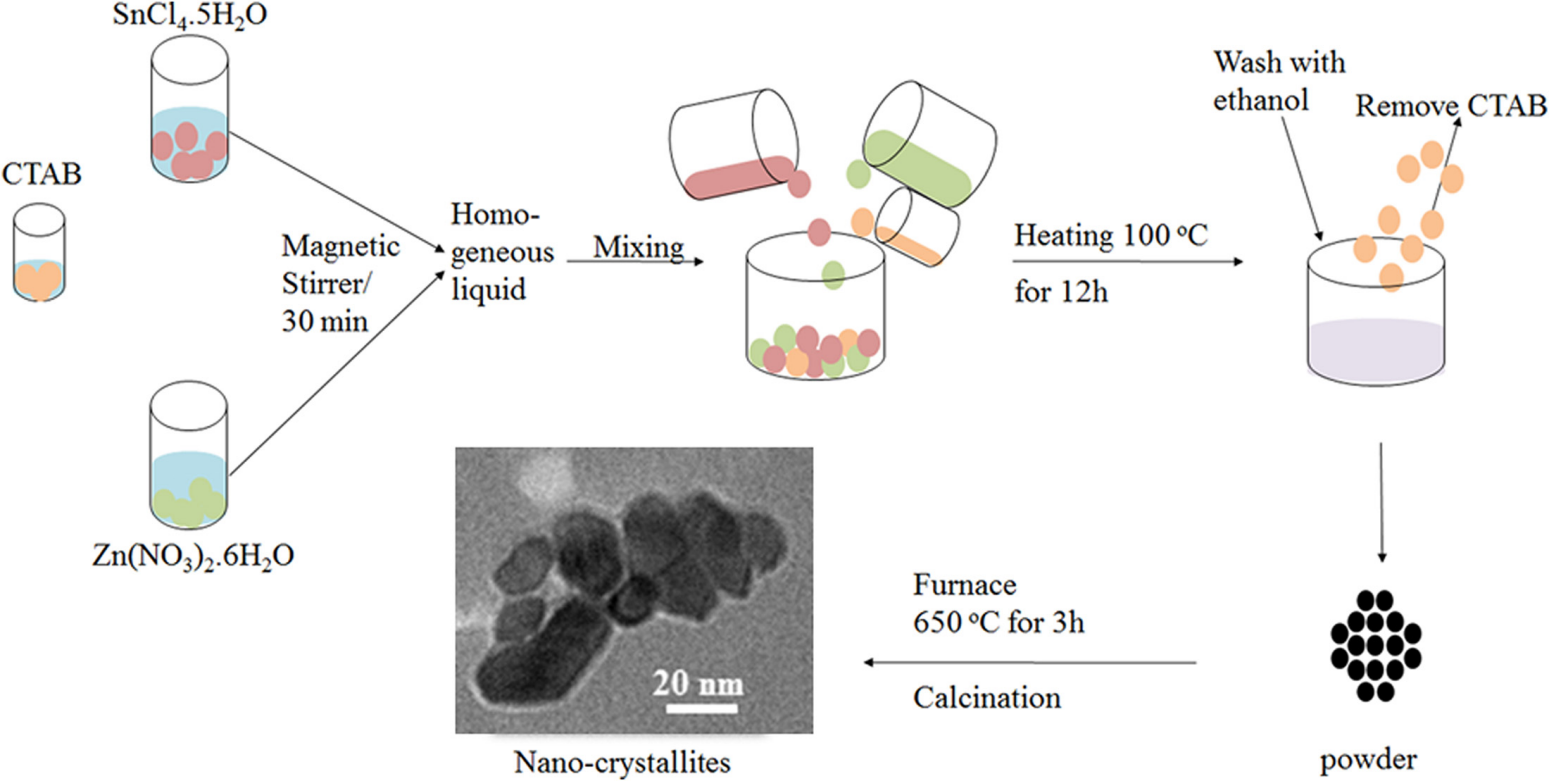
Schematic of the synthesis process.

The ZnSnO_3_, Zn_1.25_SnO_3+α,_ Zn_1.5_SnO_3+β_, Zn_1.75_SnO_3+γ_, Zn_2_SnO_4_ (where α ~ 0.25, β ~ 0.5, γ ~0.75) nano-crystallites with general formula Zn_1+x_SnO_3+x_ (0 ≤x≤ 1) have been synthesized by facile cost-effective wet chemistry route using cetyltrimethyl ammonium bromide (CTAB) as a surfactant. First, the aqueous solution of tin chloride pentahydrate (SnCl_4_.5H_2_O) and zinc nitrate hexahydrate (Zn(NO_3_)_2_.6H_2_O) in Zn/Sn molar ratio of 1, 1.25, 1.5, 1.75 and 2, respectively in 5 mM individual concentration were prepared through the consistent stirring for 30 min each in separate beakers. Subsequently, SnCl_4_.5H_2_O and Zn(NO_3_)_2_.6H_2_O solutions in desired amounts were mixed together to obtain another clear solution. Afterward, 5 mM solution of CTAB in the metal (Zn^2+^ and Sn^2+^) to CTAB molar ratio of 10:1, was added to the above solution drop-wise. These obtained five solutions were dried in oven at 100°C for 12 h, washed with ethanol afterward and subsequently dried for 6 h at 100°C, washed with ethanol again, and dried once more at 100°C for 6h. Now, these dried powders were decomposed at 650°C for 3h to obtain ZnSnO_3_, Zn_1.25_SnO_3+α,_ Zn_1.25_SnO_3+β_, Zn_1.25_SnO_3+γ_, Zn_2_SnO_4_ (where α ~ 0.25, β ~ 0.5, γ ~ 0.75) nano-crystallitess. These obtained five samples were coded as S1, S2, S3, S4 and S5, respectively for further investigation. Fig 1 shows the schematic of the synthesis process.

The phase(s) of the as-synthesized precursor powders and ZnSnO_3_, Zn_1.25_SnO_3+α,_ Zn_1.5_SnO_3+β_, Zn_1.75_SnO_3+γ_, Zn_2_SnO_4_ (where α ~ 0.25, β ~ 0.5, γ ~0.75) nano-crystallites with general formula Zn_1+x_SnO_3+x_ (0 ≤x≤ 1) were analyzed by Rigaku Miniflex X-ray diffractometer using Cu K_α1_ radiation of 1.54056 Å at 30 kV and 15 mA. Morphological characterization of samples S1 and S5 was performed by using transmission electron microscopy Hitachi (model H-7500, 120 kV) equipped with CCD Camera. Diffuse reflectance spectra of powders were recorded by UV-Visible spectrometer; PG instruments Pvt. Ltd. T90+ in the spectral range of 300–1100 nm. Photoluminescence spectra were collected in the wavelength range of 330–450 nm with the excitation wavelength of 290 nm using Simadzu RF-530 spectroflurometer. The impedance analysis at different temperatures was performed using potentiogalvanostat, Biologic SP 240, in the frequency range between 100 Hz to 3MHz. For impedance investigation, the nano-crystalline samples were pelletized in 10 mm diameter pellets at a pressure of 5 ton, sintered at 900°C for 6h, polished subsequently to 1.4 mm thickness, and silver pasted afterward. The dye-sensitized solar cell (DSSC) devices were fabricated using zinc stannate photo-anode, and cyclic voltammetry studies were performed using potentiogalvanostat, Biologic SP 240.

## Results and Discussion

### 3.1 Structural and morphological characterization

**Fig 2:**
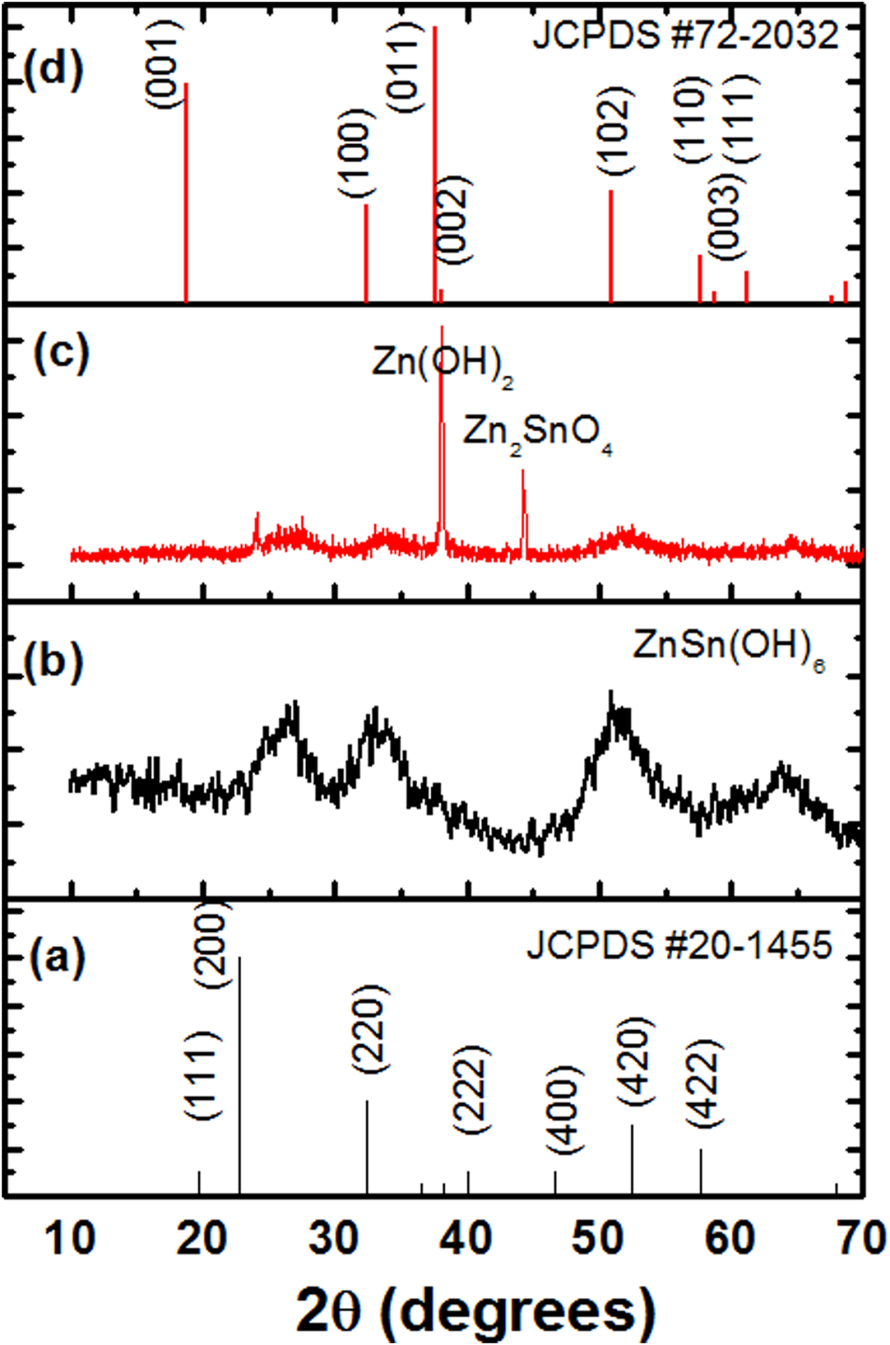
X-ray diffraction patterns of standard JCPDS cards 20–1455 and 72–2052 (a, c) and as-synthesized samples at the Zn/Sn molar ratios of 1 and 2 (b, d).

**Fig 3:**
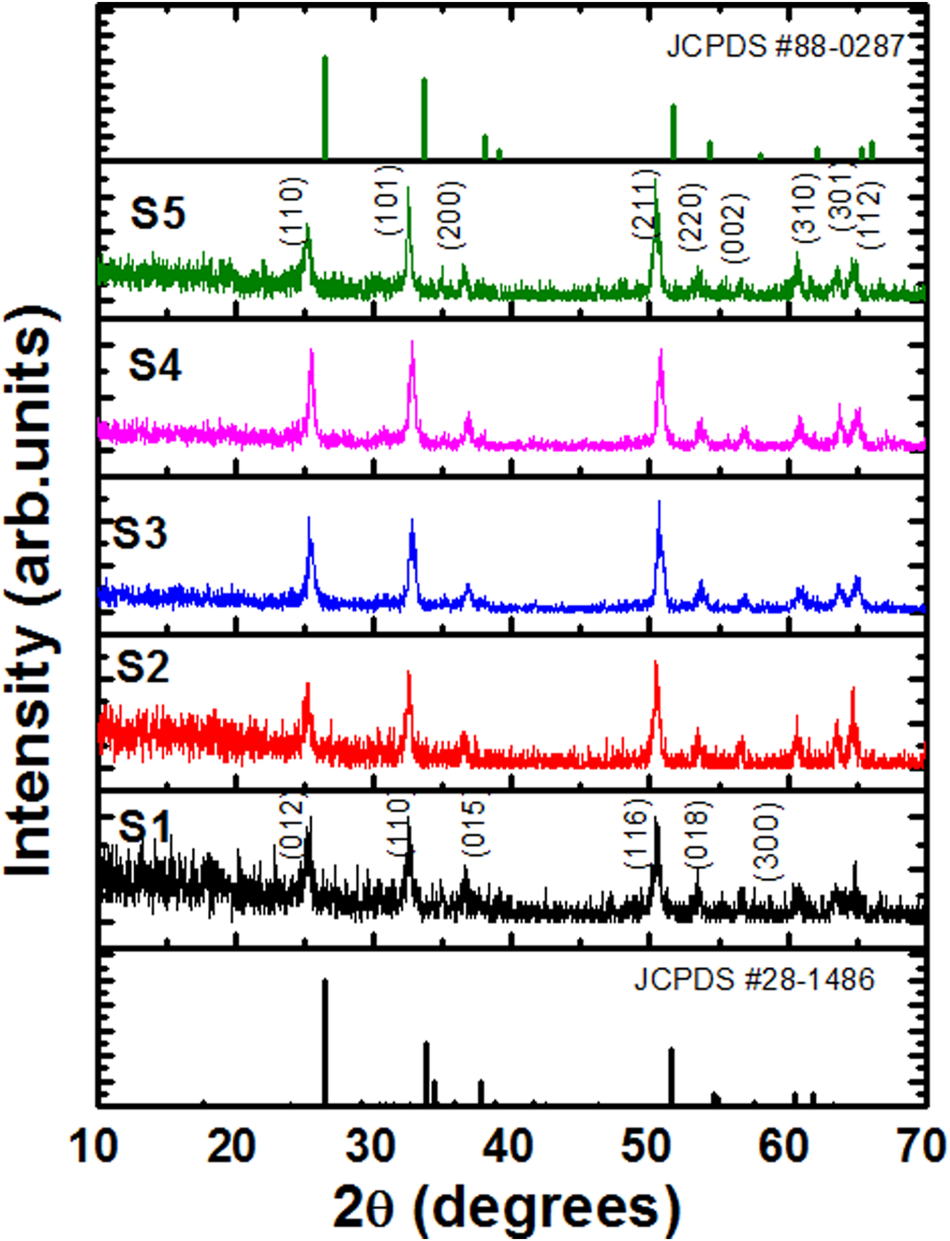
X-ray diffraction patterns of samples S1, S2, S3, S4, and S5 i.e., of the ZnSnO_3_, Zn_1.25_SnO_3+α,_ Zn_1.5_SnO_3+β_, Zn_1.75_SnO_3+γ_, Zn_2_SnO_4_ nano-crystallites, respectively obtained by decomposition of the gel product (formed through wet chemistry reaction at 100°C for 18 h) at 650°C for 3h.

Fig 2 shows the X-ray diffraction (XRD) patterns of as-synthesized samples at the Zn/Sn molar ratios of 1 and 2, respectively. The formation of single phase of ZnSn(OH)_6_ (JCPDS # 20–1455) is evident at Zn/Sn molar ratio of 1. At higher Zn/Sn content, Zn(OH)_2_ (JCPDS # 72–2032) together with ZnSn(OH)_6_ phase starts precipitating. Particularly, at the Zn/Sn molar ratio 2, small traces of Zn_2_SnO_4_ cubic phase (JCPDS # 04–736) together with ZnSn(OH)_6_ and Zn(OH)_2_ phases are also observed. The Zn(OH)_2_ crystallites are presumably oriented in [11] preferred direction as Bragg’s reflection planes other than (011) are hardly visible. The observed traces of Zn_2_SnO_4_ cubic phase too are not visible in the XRD pattern of final compound (Fig 3, discussed later). The mechanism of the reaction can be understood as follows. The SnCl_4_.5H_2_O and Zn(NO_3_)_2_.6H_2_O in aqueous solution remain dissociated as: SnCl_4_.5H_2_O → Sn^4+^ + 4Cl^-^ + 5H_2_O and Zn(NO_3_)_2_.6H_2_O→ Zn^2+^ + 2NO_3_^-^ + 6H_2_O, respectively. The Zn^2+^ and Sn^4+^ ions in Zn/Sn molar ratio of one form a hydroxide as per following reaction: Zn^2+^ + Sn^4+^ + 6OH^-^→ ZnSn(OH)_6_ [17]. On decomposition at elevated temperature, i.e., 650°C, the ZnSn(OH)_6_, forms ZnSnO_3_ as per the reaction ZnSn(OH)_6_ → ZnSnO_3_ +3H_2_O. The higher molar ratios of Zn/Sn, i.e., 1.25, 1.5, 1.75 and 2 lead to the precipitation of Zn(OH)_4_^2-^ together with the precipitation of ZnSn(OH)_6_ as per the following additional reaction, Zn^2+^ + 2OH^-^→ Zn(OH)_4_^2-^. The Zn(OH)_4_^2-^ reacts with ZnSn(OH)_6_ and forms Zn_2_SnO_4_ as per the reaction, ZnSn(OH)_6_ +Zn(OH)_4_^2-^ → Zn_2_SnO_4_ + 4H_2_O + 2OH^-^. It is obvious that when molar ratio of Zn/Sn is fractional number between 1 and 2, the precipitation of both ZnSnO_3_ and Zn_2_SnO_4_ phases will occur. The presence of the surfactant possibly alters the surface energy of the crystallites surfaces and, in turn, results in the anisotropic growth of nanoparticles. In the present work, the CTAB, which has been used as a surfactant, plays a pivotal role in monodispersion of the as synthesized nano-crystallites [27].

Fig 3 shows the X-ray diffraction patterns of the ZnSnO_3_, Zn_1.25_SnO_3+α,_ Zn_1.5_SnO_3+β_, Zn_1.75_SnO_3+γ_, Zn_2_SnO_4_ (where α ~ 0.25, β ~ 0.5, γ ~0.75) nano-crystallites obtained by decomposition of the gel product (formed through wet chemistry reaction at 100°C for 18 h) at 650°C for 3h. At Zn/Sn molar ratio of 1, it clearly indicates the formation of orthorhombic type- ZnSnO_3_ (JCPDS # 28–1486) with the values of lattice parameters being as a ~ 3.6203 Å, b ~ 4.2646 Å and c ~ 12.8291Å. On increasing the Zn/Sn molar ratio to 2, the structure revealed formation of single phase of tetragonal SnO_2_ –type Zn_2_SnO_4_ (JCPDS # 88–0287), with mixed phases of ZnSnO_3_ and Zn_2_SnO_4_ at intermediate compositions (i.e., at Zn/Sn molar ratio of 1.25, 1.50, 1.75). The crystallite sizes were estimated using the Bragg relation, i.e., D = 0.9λ/βcosθ, where D is average crystallite size, λ wavelength of the X-ray used, β, corrected full width at half maximum (FWHM) of the respective peak belonging to diffraction angle 2θ. The estimated values of crystallite size for samples S1, S2, S3, S4 and S5 were found to be 47, 58, 48, 51, and 49 nm, respectively. At Zn/Sn molar ratio of 1, the average crystallite size was 47 nm, which increased to 58 nm at Zn/Sn molar ratio of 1.25. The coordination numbers of Zn^2+^ and Sn^4+^ ions in orthorhombic ZnSnO_3_ are 4 and 6, respectively. Further, the ionic radii of Sn^4+^ and Zn^2+^ ions in six-coordination are 0.69 Å, and 0.74 Å, respectively. Obviously, the ionic radius of Zn^2+^is higher than Sn^4+^, therefore on occupying Sn^4+^ lattice sites in the ZnSnO_3_ crystal, it will put crystal under tensile stress, which, in turn, will give rise to increase crystallite size [28–29]. On increasing the Zn/Sn molar ratio to 1.5, the precipitation of secondary phase, i.e., Zn_2_SnO_4_ becomes significant to inhibit the growth of primary phase crystallites, i.e., ZnSnO_3_. Due to this reason the crystallite size of sample S3 has been found to be smaller than the sample S2. At Zn/Sn molar ratio of 1.75, i.e., for sample S4, the size increased slightly in comparison to sample S3, which may be due to the fact that sample S4 at Zn/Sn molar ratio of 1.75 will have higher amount of tetragonal Zn_2_SnO_4_ phase formation than sample S3. In contrary to sample S2, where primary phase was orthorhombic type- ZnSnO_3_, in this case, the primary phase is Zn_2_SnO_4_. As discussed before, in case of sample S2, the lower amount of Zn_2_SnO_4_ phase was not able to inhibit the growth of ZnSnO_3_. Similarly in present case the lower amount of ZnSnO_3_ phase is not able to inhibit the growth of Zn_2_SnO_4_ crystallites, and, in turn, exhibit increased crystallite size than sample S3. Further increase in Zn/Sn molar ratio (Zn/Sn = 2) reveals a slight decrease in crystallite size (~ 49 nm), possibly due to inherent oxygen vacancies in Zn_2_SnO_4_, which, put crystal under compressive stress. The values of lattice parameters for tetragonal Zn_2_SnO_4_ were as a = b ~ 5.0136 Å and c ~3.3055Å.

**Fig 4:**
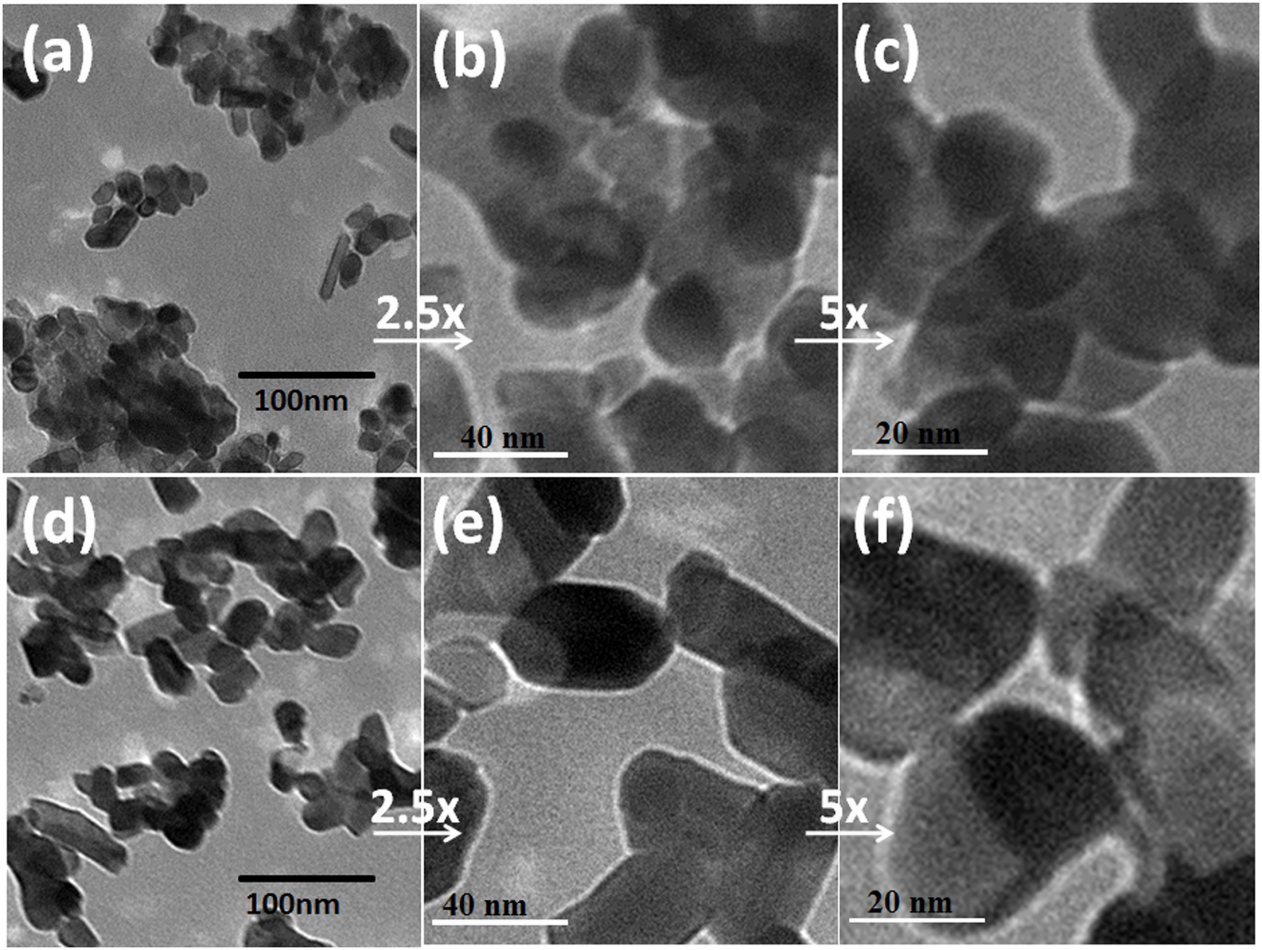
(a-c) transmission electron micrographs of ZnSnO_3_ (S1) and (d-f) of Zn_2_SnO_4_ (S5) samples.

Fig 4 shows transmission electron micrographs of ZnSnO_3_ and Zn_2_SnO_4_ samples, i.e., for samples S1 and S5. The sample S1 exhibits nano-rod type morphology with average aspect ratio of ~2; the length and thickness being as ~24 nm and ~13 nm, respectively. The sample S5 retains the similar morphology; the average crystallite length being ~ 47 nm and thickness ~22 nm, respectively. The average aspect ratio remains almost same. It is obvious that crystallite size obtained via transmission electron microscopy (TEM) observations are smaller than that of estimated from X-ray diffraction line broadening using Scherrer equation. The possible reason may be settling down of large particles during TEM sample preparation.

### 3.2 Optical characterization

S1 Fig shows diffused reflectance spectra of the samples S1, S2, S3, S4 and S5, i.e., ZnSnO_3_, Zn_1.25_SnO_3+α,_ Zn_1.5_SnO_3+β_, Zn_1.75_SnO_3+γ_, Zn_2_SnO_4_ (where α ~ 0.25, β ~ 0.5, γ ~0.75) nano-crystallites obtained by decomposition of the gel product (formed through wet chemistry reaction at 100°C for 18 h) at 650°C for 3h. As per optical absorbance and Kubelka-Munk function, the pure diffuse reflectance of the sample can be expressed as [30],

$$\text{F}\left({\text{R}}_{\text{pd}}\right)\;=\;{\left(1-{\text{ R}}_{\text{pd}}\right)}^{2}/\;2{\text{ R}}_{\text{pd}}=\text{ K}/\text{S}$$ (1)

where R_pd_ is the pure diffuse reflectance, K is absorption coefficient and S is scattering coefficient. The pure diffuse reflectance, F(R_pd_) is proportional to the molar absorption coefficient (α). The relation between optical bandgap (E_g_) and α can be given by well-known Tauc relation [30],

$$(\alpha \text{h}\upsilon )\;=\text{C}{\left(\text{h}\upsilon -{\text{E}}_{\text{g}}\right)}^{\text{n}}$$ (2)

where *hυ* is the energy of the absorbed photon, and C is proportionality constant. Also, from Eqs 1 and 2, following relations can be obtained [31–33],

$$[(\text{F}\left({\text{R}}_{\text{pd}}\right)\text{h}\upsilon ]\;=\text{C}{\left(\text{h}\upsilon -{\text{E}}_{\text{g}}\right)}^{\text{n}}$$ (3)

where n equals to ½ for allowed direct transition, 1 for non-metallic materials, 3/2 for direct forbidden transitions, 2 for allowed indirect transitions and 3 for indirect forbidden transitions, respectively [31–33]. For estimation of bandgap values, the [(F(R_pd_)hυ]^1/n^ versus hυ plots are extrapolated to linear fitted region at [(F(R_pd_)hυ]^1/n^ = 0.

**Fig 5:**
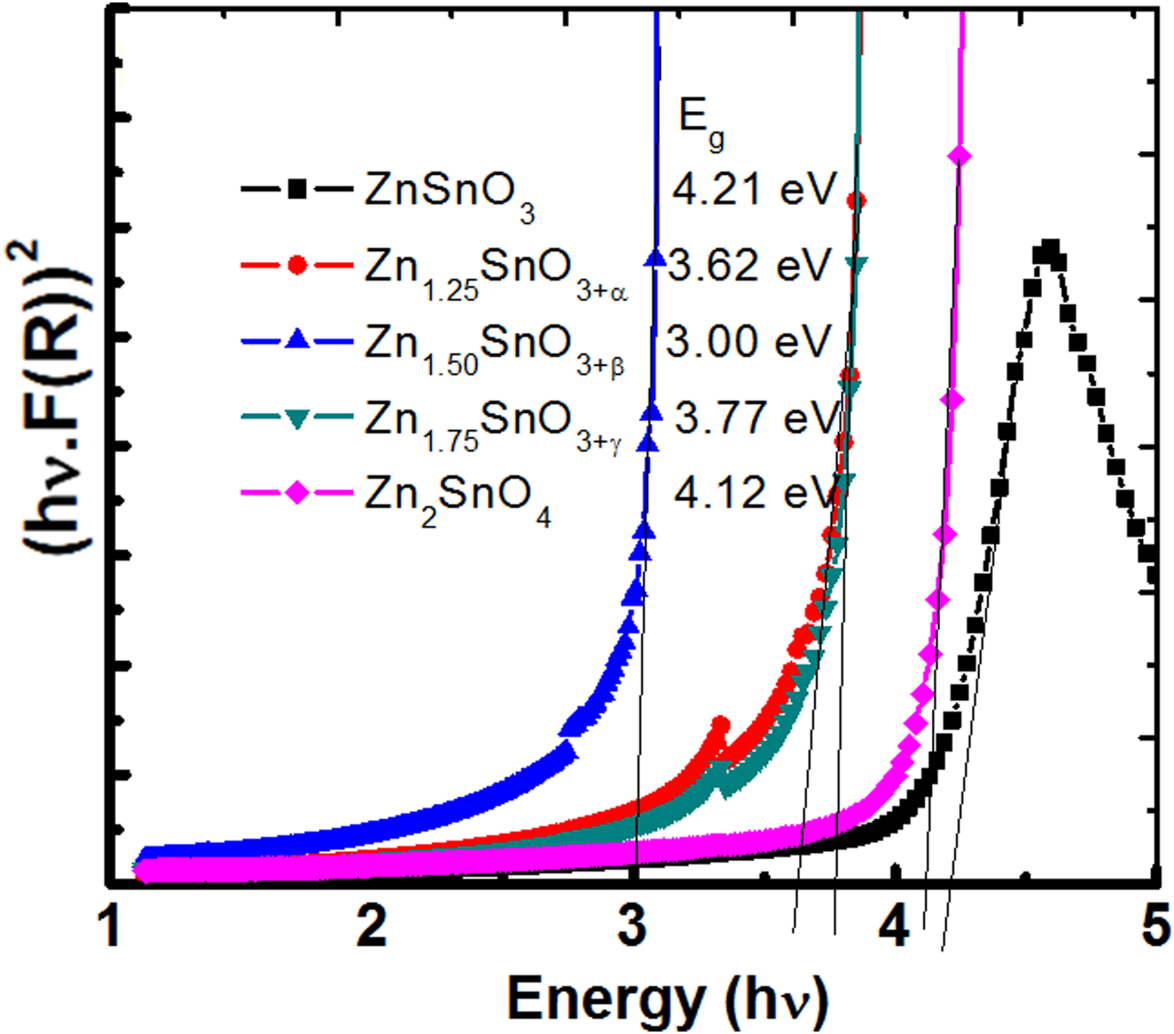
Band gap estimation of ZnSnO_3_, Zn_1.25_SnO_3+α,_ Zn_1.5_SnO_3+β_, Zn_1.75_SnO_3+γ_, Zn_2_SnO_4_ nano-crystallites from diffused reflectance spectra (S1 Fig) obtained by decomposition of the gel product at 650°C for 3h.

The ZnSnO_3_ and Zn_2_SnO_4_ are known to be direct wide bandgap semiconductors [34–38]. Therefore, direct bandgap values have been estimated using Eq 3, absorption data of S1 Fig and are shown in Fig 5. The estimated values of bandgap are 4.21, 3.62, 3.00, 3.77 and 4.12 eV for samples S1, S2, S3, S4 and S5, i.e., ZnSnO_3_, Zn_1.25_SnO_3+α,_ Zn_1.5_SnO_3+β_, Zn_1.75_SnO_3+γ_, Zn_2_SnO_4_ (where α ~ 0.25, β ~ 0.5, γ ~0.75) nano-crystallites, respectively. The reported values of bandgap for ZnSnO_3_ and Zn_2_SnO_4_ bulk samples are 3.4–3.9 eV [39–41] and 3.3 eV [41], respectively. Not many reports are available on the bandgap studies of ZnSnO_3_, and further the absorbance data of Zn_2_SnO_4_ have been debated [34–38]. Therefore, these samples have further been investigated using photoluminescence studies.

**Fig 6:**
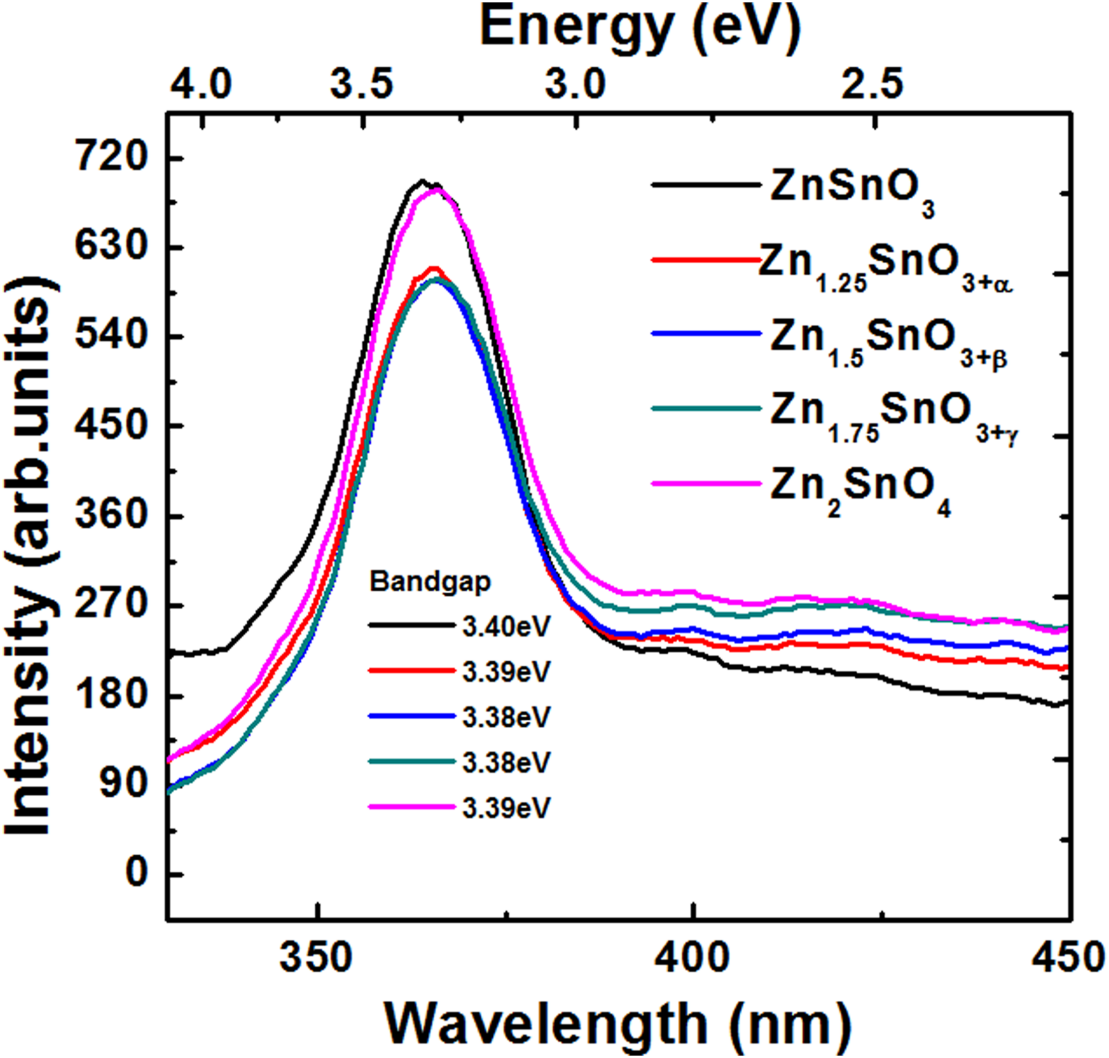
Emission spectra of ZnSnO_3_, Zn_1.25_SnO_3+α,_ Zn_1.5_SnO_3+β_, Zn_1.75_SnO_3+γ_, Zn_2_SnO_4_ obtained at the excitation wavelength of 290 nm.

Fig 6 show the emission spectra of the samples S1, S2, S3, S4 and S5, i.e., ZnSnO_3_, Zn_1.25_SnO_3+α,_ Zn_1.5_SnO_3+β_, Zn_1.75_SnO_3+γ_, Zn_2_SnO_4_ obtained at the excitation wavelength of 290 nm. It is evident that in case of sample S1, i.e. ZnSnO_3_, the emission peak occurs at the wavelength of around 365 nm (energy ~ 3.40 eV). The increase in Zn/Sn molar ratio to 1.25, leads to slight redshift in peak energy ~ 3.39 eV, and at Zn/Sn molar ratio of 1.5 to 3.38 eV. Afterward, it retains its position till Zn/Sn molar ratio to 1.75. At Zn/Sn molar ratio of 2, the peak exhibits a little blue-shift in energy ~ 3.39 eV.

In fact samples S2, S3 and S4 are dual phase composites of the nano-crystalline ZnSnO_3_, and Zn_2_SnO_4_ semiconductors. Assuming that at Zn/Sn ratio of 1, 1.25, 1.5, 1.75 and 2, the precipitated phases are of pure ZnSnO_3_, and Zn_2_SnO_4_ nano-crystalline semiconductors only, the ratios of ZnSnO_3_ to Zn_2_SnO_4_ phases at Zn/Sn molar ratios of 1, 1.25, 1.5, 1.75 and 2, should be 1::0, 0.75::0.25, 0.5::0.5, 0.25::0.75, and 0::1, respectively. Following the simple mixture rule the estimated bandgap values should be as 4.21, 4.19, 4.17, 4.14 and 4.12 eV, respectively (as the estimated bandgap values for semiconductors ZnSnO_3_, and Zn_2_SnO_4_ are 4.21 and 4.12 eV, respectively). The deviation in estimated bandgap values is substantial for intermediate composite samples, i.e., samples S2, S3 and S4. Further, the emission spectra do not show such drastic variation in bandgap. It indicates that somehow the diffused reflectance curves of intermediate samples which results due to overlapping of two phase compounds propagate more errors in bandgap estimation, i.e., the assumption of considering the intermediate samples as single phase and estimation of the bandgap does not fits well. As discussed before, the fact of dual phase formation in intermediate compounds has been verified through XRD investigation and is shown in Fig 3. To understand the these samples further, the excitation spectra of samples S1 and S5 were collected at the emission wavelength of 366 nm and are shown in S2 Fig. These show excitation at almost same wavelength as emission; indicating direct bandgap semiconductor nature of these samples. It is obvious that photoluminescence investigation (emission and absorption spectra) is more close to experimentally reported bandgap values [39–41] in comparison to estimated bandgap values using diffused reflectance spectra.

### 3.3 Variation of ac conductivity, permittivity and loss tangent

**Fig 7:**
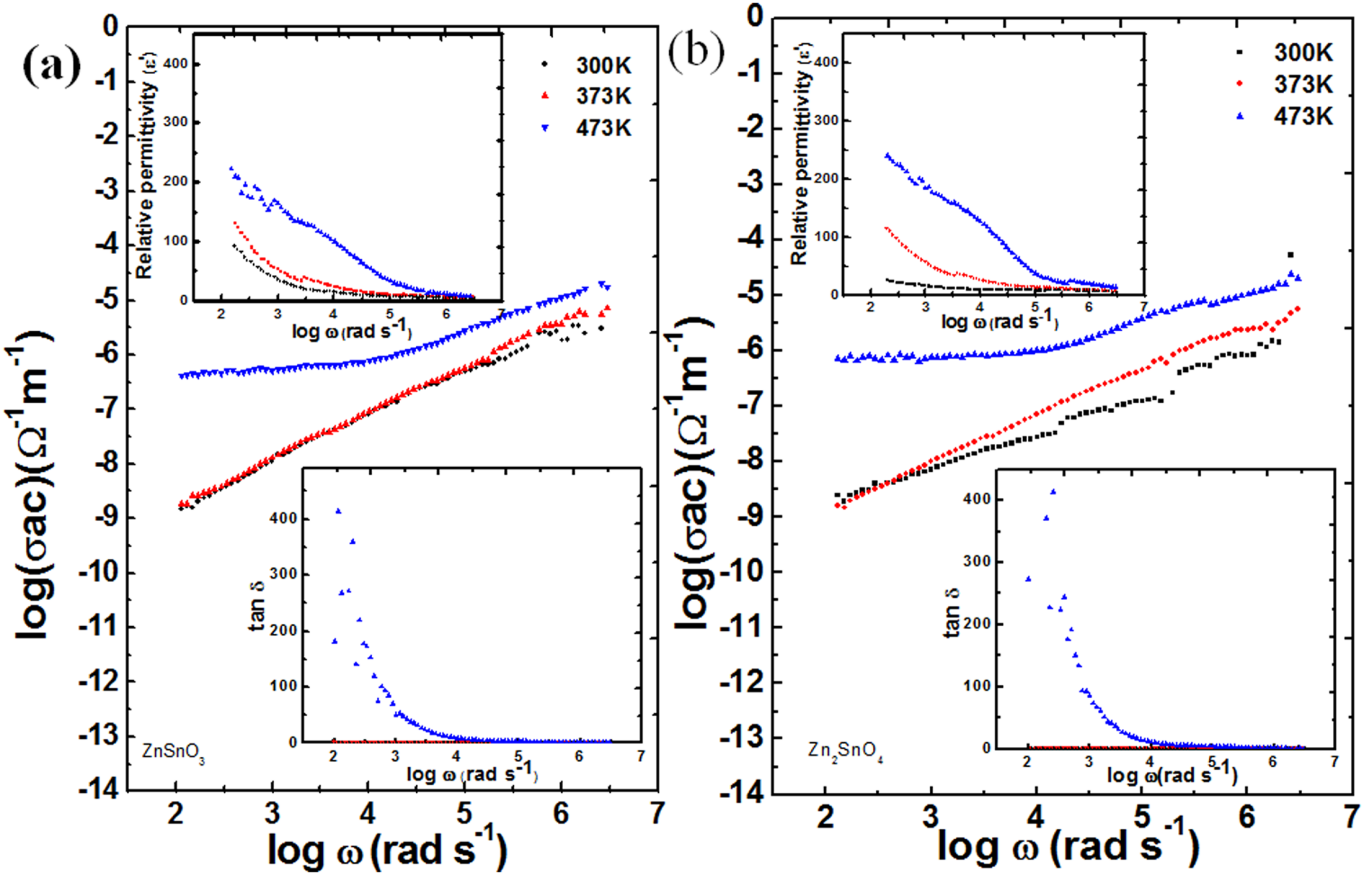
(a, b) variation of ac conductivity with frequency at temperature range up to 473 K, top left corner inset shows variation of relative permittivity with frequency for samples ZnSnO_3_ (Fig 7a) and Zn_2_SnO_4_ (Fig 7b), bottom right corner insets show loss tangent as the function of frequency for ZnSnO_3_ and Zn_2_SnO_4_, respectively.

Fig 7(a) and 7(b) shows variation of ac conductivity with frequency in the temperature range up to 473 K. The conductivity variation as a function of temperature can be shown as σ (ω) = ωε_o_ε’tan(δ) [42], where ε_o,_ ε’, and tan(δ) are permittivity of free space, relative permittivity of the sample, and loss tangent, respectively at the frequency ω. Obviously, the conductivity increases with frequency as well as with temperature (Fig 7a and 7b) for both the samples ZnSnO_3_ and Zn_2_SnO_4_. The ac conductivity values for the sample ZnSnO_3_ at the frequency of 10 kHz are 8.02×10^−8^Ω^-1^ cm^-1^, 8.72×10^−8^Ω^-1^ cm^-1^ and 7.68×10^−7^Ω^-1^ cm^-1^ for the temperature of 300, 373 and 473 K, respectively. It is evident that the conductivity values increase with the increase in temperature as more charge carriers become available for conduction at increased temperature. The ac conductivity values for Zn_2_SnO_4_ nano-crystalline sample at the frequency of 10 kHz were found to be 6.77×10^−8^ Ω^-1^ cm^-1^, 8.06×10^−8^ Ω^-1^ cm^-1^ and 1.00×10^−6^ Ω^-1^ cm^-1.^ Clearly, the conductivity values for the sample Zn_2_SnO_4_ are lower than ZnSnO_3_ upto the temperature of 373 K, and subsequently become higher at the temperature of 473 K. This may be ascribed to its different crystal structure (crystal structure of Zn_2_SnO_4_ and ZnSnO_3_ are tetragonal and orthorhombic, respectively), which leads to different crystal defects. The number of oxygen defects is possibly higher in Zn_2_SnO_4-Δ_, (where Δ shows oxygen defects) in comparison to ZnSnO_3-▲_ (where ▲ shows oxygen defects) samples, which, in turn lead to higher charge carrier density at elevated temperatures. This fact has also been discussed in structural analysis Section 3.1 for the explanation of reduced crystallite size at the Zn/Sn ratio of 2.

Top left corner insets of Fig 7(a) and 7(b) show variation of relative permittivity with frequency for samples ZnSnO_3_ and Zn_2_SnO_4_^,^ respectively. Both of these samples exhibit a plateau region; and clear dispersive nature at lower frequencies. Particularly, at 10 kHz the values of relative permittivity for sample ZnSnO_3_ are 14.24, 20.83 and 103.90 at the temperature of 300, 373 and 473 K, respectively. The values of relative permittivity for Zn_2_SnO_4_ sample are higher than ZnSnO_3_ samples at the same temperature values, and have been observed as 25.33, 34.00, and 124.61, respectively. The relative permittivity decreases with increase in frequency due to deformation and relaxation polarization. The deformation and relaxation depend on displacement and orientation of charge carriers, respectively. Moreover, at higher frequencies molecular dipoles take more time to reorient and thereby lead to decrease in orientation polarization. The relative permittivity values for Zn_2_SnO_4_ sample are higher than ZnSnO_3_ samples. Also, as discussed before, optical absorbance studies revealed that the estimated bandgap values for the samples ZnSnO_3_ and Zn_2_SnO_4_ as 4.21 and 4.12 eV (direct bandgap), respectively. According to electric polarizability phenomenon the decrease in bandgap leads to increase in the values of the relative permittivity.

Insets of Fig 7(a) and 7(b), at bottom right corner, shows the variation of loss tangent as the function of frequency for the samples ZnSnO_3_and Zn_2_SnO_4_, respectively. The loss tangent values for the sample ZnSnO_3_ are 0.89, 0.92, and 8.81, respectively at the temperature of 300, 373 and 473 K, respectively. The values of loss tangent for the sample Zn_2_SnO_4_ are 0.72, 0.86 and 10.15, respectively at the temperature of 300, 373 and 473 K, respectively. The values of loss tangent for both the samples at 473 K are increased considerably due to increased phonon energy at elevated temperatures caused by increased thermal vibrations.

### 3.4 Cyclic voltammetry of dye-sensitized solar cell

**Fig 8:**
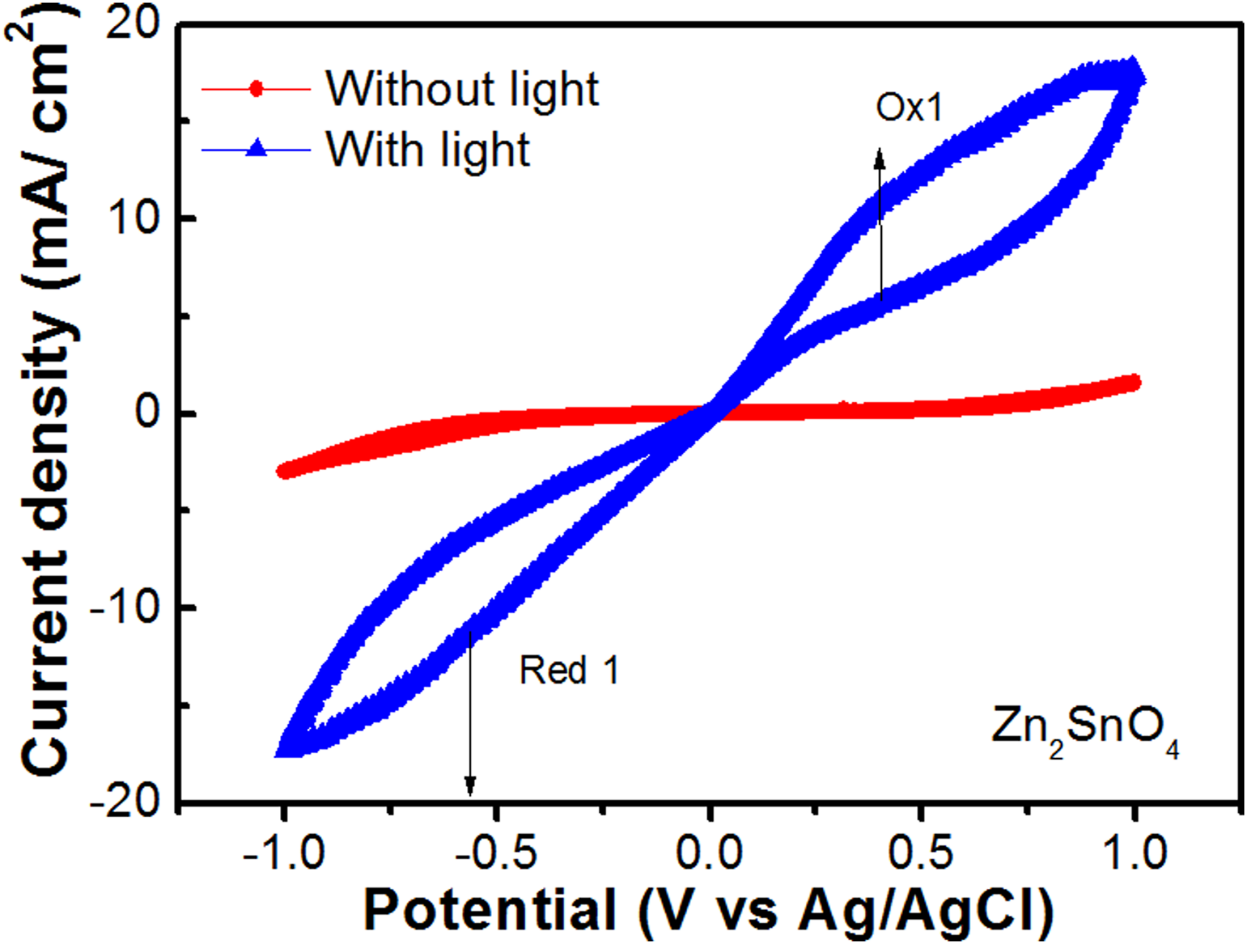
Electron-transfer properties of the electrode using cyclic voltammetry (CV) in I^–^/I3– electrolyte composition with Zn_2_SnO_4_ photo-anode.

Fig 8 shows cyclic voltammogram of the typical DSSC device (in light and no light conditions) of area 4×6 mm^2^ fabricated using ITO coated glass, zinc stannate photo-anode (Zn_2_SnO_4_), platinum counter electrode, I^–^/I_3_^–^ electrolyte, and N749 dye. The I^–^/I_3_^–^electrolyte was synthesized by using Imidazolium iodide 0.7 M (0.8824 g) I_2_ 0.03 M (0.0381g), GSCN (Guanidine thiocyanate), 0.05 M (0.0305 g), TBP (4-tert-butylpyridine), 0.5 M (0.3414 g) and ACN/VN (acetonitrile/valeronitrile) (85:15), 4.25 ml: 0.75 ml. In absence of light, oxidation (3I^–^ → I_3_^–^ + 2e) or reduction (I_3_^–^ + 2e → 3I^–^) peaks are not clearly visible, which indicates that there is no sufficient change transfer from platinum electrode. However, in light conditions there is more obvious change transfer. The oxidation and reduction peaks are observed at 0.40 V (current density ~ 11.1 mA/cm^2^) and, 0.57 V (current density– 11.7 mA/cm^2^), respectively; indicating good performance of the device. The cyclic voltammetry (CV) is a very versatile technique to investigate electron-transfer properties of the electrodes. Lan et. al. [43] used CV to compare the catalytic activity of various counter electrodes used is DSSC.

## Conclusions

The cetyltrimethyl ammonium bromide (CTAB) stabilized Zn_1+x_SnO_3+x_ (0 ≤ x ≤1) nano-crystallites have successfully been synthesized using facile cost-effective wet chemistry route. The structural analysis confirmed the formation of orthorhombic ZnSnO_3_, tetragonal SnO_2_-type Zn_2_SnO_4_ and/or their mixed phases depending upon the Zn/Sn ratio of the precursors. The morphological analysis exhibited ZnSnO_3_ crystallites to be approximately of the half size that of Zn_2_SnO_4_ crystallites (the length and thickness being 24, 13 for ZnSnO_3_ and 47, 22 nm for Zn_2_SnO_4_), respectively with aspect ratio of 2. The UV-visible diffuse reflectance together with photoluminescence data revealed these nano-crystallites to be direct wide bandgap semiconductors. The emission spectra exhibited bandgap of these nano-crystallites to be ~3.40 eV. Further, the room temperature ac conductivity values for the ZnSnO_3_ were found to be higher than Zn_2_SnO_4_ samples. The cyclic voltammetry analysis of ZnSnO_3_ and Zn_2_SnO_4_ photo-anode based dye-sensitized solar cell revealed oxidation and reduction around 0.40 V and, 0.57V, respectively.

## Supporting Information

S1 Fig Diffused reflectance spectra of ZnSnO_3_, Zn_1.25_SnO_3+α,_ Zn_1.5_SnO_3+β_, Zn_1.75_SnO_3+γ_, Zn_2_SnO_4_ nano-crystallites obtained by decomposition of the gel product at 650°C for 3h. (TIF)

S2 Fig Excitation spectra of ZnSnO_3_ and Zn_2_SnO_4_ nano-crystallites at the emission wavelength of 366 nm. (TIF)

S1 File Data of Fig 2. (XLSX)

S2 File Data of Fig 3. (XLSX)

S3 File Data of Fig 6. (XLSX)

S4 File Data of Fig 7. (XLSX)

S5 File Data of Fig 8. (XLSX)
