# Supplementary figures and images for: Synthesis and Behavior of Cetyltrimethyl Ammonium Bromide Stabilized Zn1+xSnO3+x (0 ≤ x ≤1) Nano-Crystallites
Source: PLoS One. 2016 May 26;11(5):e0156246. doi: 10.1371/journal.pone.0156246 (PMC4881896; doi:10.1371/journal.pone.0156246)

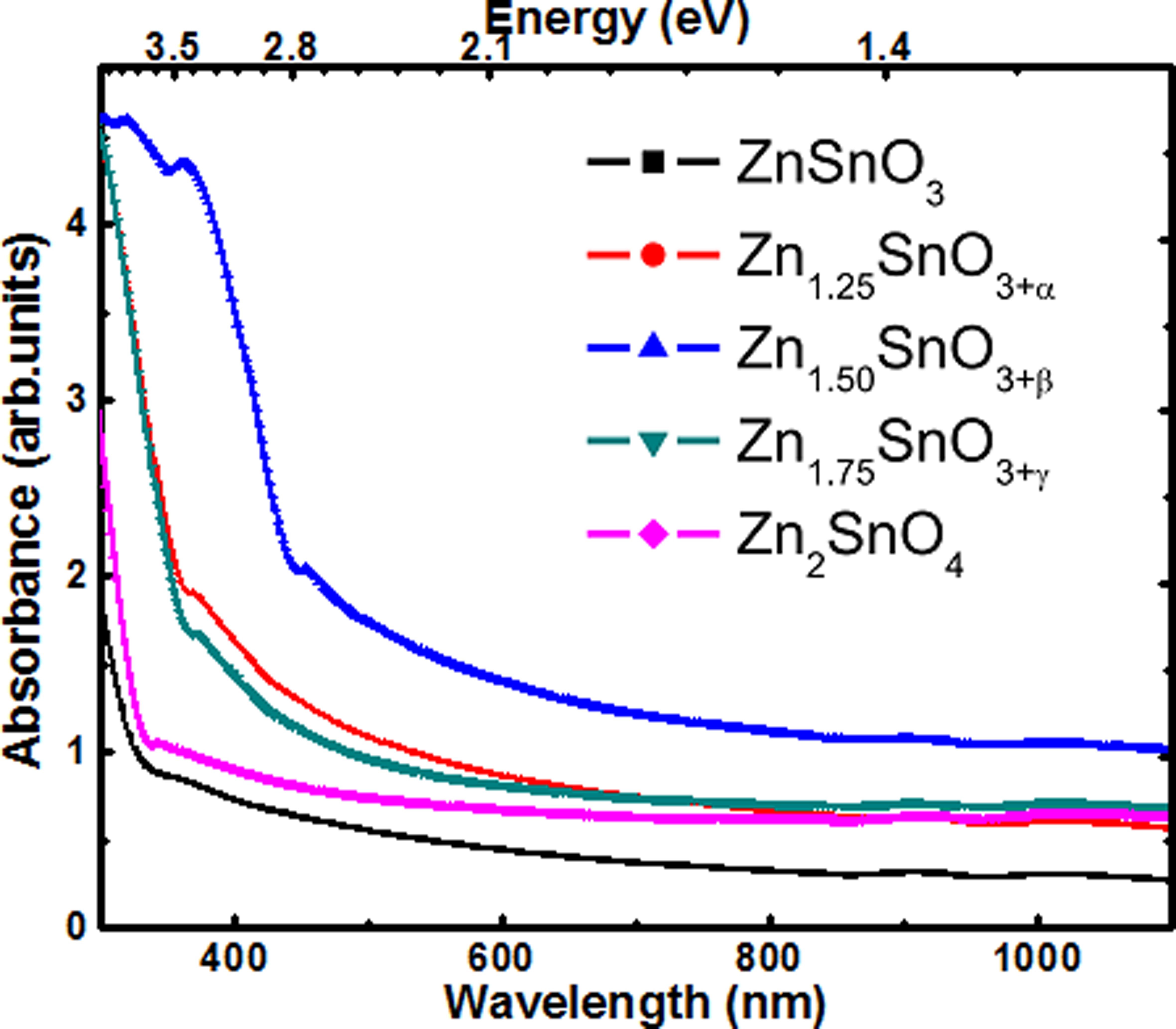

Supplement: S1 Fig — (TIF) [file pone.0156246.s001.tif]

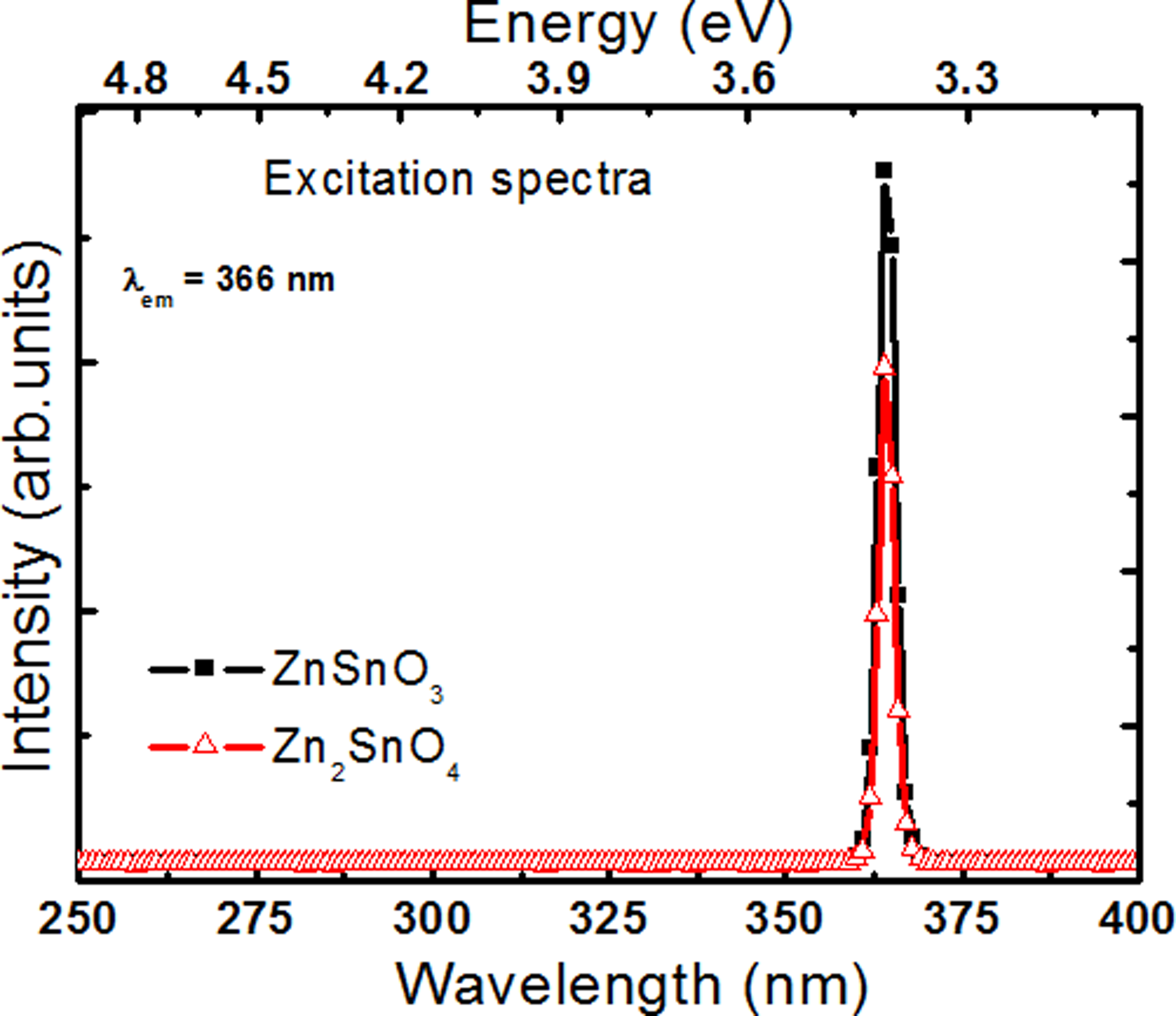

Supplement: S2 Fig — (TIF) [file pone.0156246.s002.tif]
